# Atomistic Theory of Hot-Carrier Relaxation in Large Plasmonic Nanoparticles

**DOI:** 10.1021/acs.jpcc.3c05347

**Published:** 2023-11-23

**Authors:** Simão M. João, Hanwen Jin, Johannes C. Lischner

**Affiliations:** Department of Materials, Imperial College London, South Kensington Campus, London SW7 2AZ, U.K.

## Abstract

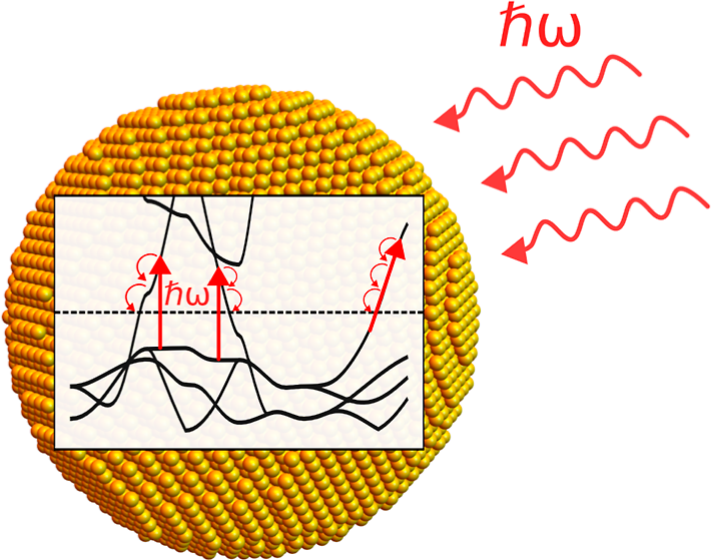

Recently, there has been significant interest in harnessing hot-carriers generated from the decay of localized surface plasmons in metallic nanoparticles for applications in photocatalysis, photovoltaics, and sensing. In this work, we develop an atomistic method that makes it possible to predict the population of hot-carriers under continuous wave illumination for large nanoparticles of relevance to experimental studies. For this, we solve the equation of motion of the density matrix, taking into account both the excitation of hot-carriers and subsequent relaxation effects. We present results for spherical Au and Ag nanoparticles with up to 250,000 atoms. We find that the population of highly energetic carriers depends on both the material and the nanoparticle size. We also study the increase in the electronic temperature upon illumination and find that Ag nanoparticles exhibit a much larger temperature increase than Au nanoparticles. Finally, we investigate the effect of using different models for the relaxation matrix but find that the qualitative features of the hot-carrier population are robust. These insights can be harnessed for the design of improved hot-carrier devices.

## 1 Introduction

Metallic nanoparticles (NPs) feature strong and highly tunable light–matter interactions, which makes them attractive for applications in photovoltaics and photocatalysis.^1−5^ In particular, such NPs exhibit collective charge oscillations known as localized surface plasmons (LSPs),^6^ which give rise to a pronounced peak in the optical absorption spectrum at the localized surface plasmon resonance (LSPR) frequency. LSPs are strongly damped oscillations that can decay via the Landau damping mechanism into energetic electron–hole pairs known as hot-carriers, which can then be harnessed for different applications.

After their generation,^7^ hot-carriers lose their energy as a result of interactions with other electrons, lattice vibrations,^3,8,9^ or defects. In addition, they can also interact with molecular species adsorbed to the surface of the NP, which in turn can induce adsorbate vibrations, desorption events, or chemical transformations. The transfer of energy from the electrons to the lattice leads to an increase in the lattice temperature. Such plasmonic heating can be used for photothermal applications (which typically do not require the extraction of hot-carriers) but also plays an important role in catalysis.^10−12^ A detailed understanding of how these various processes influence and depend on the population of hot-carriers is important for optimizing devices.

To gain microscopic insights into hot-carrier relaxation processes, various theoretical approaches have been developed. For example, Dubi and Sivan^10^ solved the Boltzmann equation to calculate the steady-state population of hot-carriers, taking both electron–electron and electron–phonon scattering effects into account. A fully quantum-mechanical approach based on the equation of motion for the electronic density matrix was developed by Govorov and co-workers,^13,14^ who employed a relaxation time approximation. Another way to include relaxation effects is by solving a master equation for the electronic occupations of the NP states. Electron–phonon, electron–electron, and electron–photon scattering mechanisms can be included and their relative importance estimated.^15,16^

While the aforementioned modeling efforts allowed important insights into the relaxation dynamics of hot-carriers, it is important to note that they all employed highly simplified models of the NP electronic structure: they often use simple spherical well models or assume that the NP’s density of states is the same as that of a bulk-free electron gas. As a consequence, such models cannot capture hot-carriers generated in d bands,^17^ describe the dependence of the electronic structure on the exposed facets of the NP,^18^ or describe plasmonic heterostructures atomistically,^19^ all of which can play a critical role.^20−24^

In this paper, we start from the density matrix formalism developed in ref (13) and combine it with an atomistic model of the NP’s electronic structure based on the tight-binding approach. To enable the simulation of large NPs, we cast the expression of the hot-carrier population in a basis-independent way and use the Kernel Polynomial Method (KPM)^25,26^ to evaluate it. In recent years, KPM and other spectral methods have been established as promising tools to study quantum-mechanical phenomena in mesoscopic systems. For example, they have been used to extract spectral properties^27−29^ and transport properties^30−36^ in multibillion atom lattices. Recently, these methods have also been leveraged to obtain the rate of hot-carrier generation in million-atom plasmonic NPs^37^ and were successfully used to explain differences in photocatalytic performance of various Au–Pd nanoarchitectures^19^ and Au NP shapes.^38^ Here, we use this approach to study the hot-carrier population in large spherical Au and Ag NPs.

The paper is organized as follows. In Section 2, we explain how the optical properties of the NP, the electronic Hamiltonian, and the density matrix are constructed before using these quantities to determine the nonequilibrium steady-state hot-carrier population. In Section 3, we discuss the sensitivity of the hot-carrier population to the values of the relaxation times and then study the dependence of the hot-carrier population on the diameter of spherical Au and Ag NPs. This section ends with a discussion of the temperature increase in illuminated NPs.

## 2 Theory and Computational Details

In this section, we describe our theoretical and computational methodology. For further details, see ref (37). We start with the optical properties of the NP, discuss the electronic Hamiltonian and density matrix, and finally show how the steady-state population can be obtained in a basis-independent way.

### 2.1 Optical Properties

The interaction of the NP with light is modeled by considering the effect of a monochromatic external electric field of angular frequency ω given by , where *k* denotes the wave vector and *E*_0_ is the amplitude. In this paper, we are interested in NPs whose diameters range from ∼2 to ∼20 nm, which are much smaller than the typical wavelength of the external electric fields. In this case, **E**_ext_ can be considered uniform inside the NP. This is the quasistatic approximation. To calculate the total electric field experienced by the electrons inside the NP, it is then sufficient to solve Laplace’s equation ∇·(ε(**r**,ω)∇ϕ(**r**,ω)) = 0 for the total electric potential ϕ, with a boundary condition at infinity ϕ(**r**,*t*) = −*E*_0_*z* cos(ω*t*) and appropriate dielectric functions ε(ω) in each region. Here, we are only considering spherical NPs, for which the analytical solution of Laplace’s equation is given by
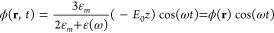
where ε_*m*_ is the dielectric constant of the environment and the measured dielectric function of the bulk material (either Ag or Au) was taken from ref (39). Here, we consider NPs in a vacuum and set ε_*m*_ = ε_0_, the vacuum permittivity. The factor
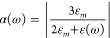
1reflects the enhancement of the electric field inside the NP. The potential energy Φ(**r**,*t*) of an electron at position **r** and time *t* is given by Φ(**r**,*t*) = −*e*ϕ(**r**,*t*), where −*e* < 0 is the electron’s charge. The excited electron–hole pairs then interact with phonons and other electrons and relax to a nonequilibrium population under continuous wave illumination.

### 2.2 Electronic Hamiltonian

The dynamics of electrons is described by a tight-binding model *H* = *H*_0_ + Φ(*t*), where
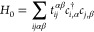
represents the Hamiltonian in the absence of illumination and
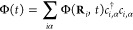
describes the optical perturbation. Here, *c*_*i*,α_^†^ (*c*_*i*,α_) denotes the creation (annihilation) operator of an electron in orbital α of an atom at position **R**_*i*_, and *t*_*ij*_^αβ^ is the hopping and onsite matrix. The double sum over *i* and *j* is typically constrained to a small number of neighbors, making *t*_*ij*_^αβ^ a sparse matrix. The tight-binding models used in this paper are obtained through an orthogonal two-center Slater-Koster parameterization.^40^ For Au, we use a parameterization consisting of 5d, 6s, and 6p atomic orbitals, and for Ag, we use 4d, 5s, and 5p orbitals.

### 2.3 Density Matrix

To determine the nonequilibrium population of electrons inside the NP, we solve the equation of motion for the electronic density matrix ρ(*t*) given by^13^
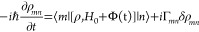
where |*n*⟩ denotes an eigenstate of *H*_0_ with corresponding eigenenergy ε_*n*_ and ρ_*mn*_ = ⟨*m*|ρ|*n*⟩. Relaxation effects are described phenomenologically through matrix Γ_*mn*_. Also, δρ_*mn*_ = ρ_*mn*_ – ρ_*mn*_^0^, where ρ_*mn*_^0^ = δ_*nm*_*f*(ε_*n*_) is the density matrix before the perturbation has been turned on, given by the Fermi–Dirac distribution , with chemical potential μ and β = 1/(*k*_B_*T*) (with *k*_B_ and *T* denoting the Boltzmann constant and the temperature, respectively). We assume that *T* corresponds to the temperature of the NP environment since the system will relax to this temperature over sufficiently long time scales. We, therefore, used room temperature (*T* = 298 K) in all of our calculations. An alternative choice for *T* would be the temperature of the NP lattice. However, the lattice heats up when the NP is illuminated, and therefore, the determination of *T* would require a self-consistent calculation. We also note that the material-specific parameters in the equation of motion for the density matrix depend on temperature in principle. However, we only consider relatively weak illumination intensities, such that this effect can be neglected.

The equation of motion for the density matrix can be solved using perturbation theory, assuming a weak monochromatic perturbation Φ(*t*) = φ_ω_e^*i*ω*t*^ + φ_ω_^†^e^–*i*ω*t*^.^13^ The result can be used to calculate the excess density of electrons with energy *E*, δ*N*(*E*) = ∑_*n*_δρ_*nn*_δ(*E* – ε_*n*_), relative to the equilibrium value. This yields
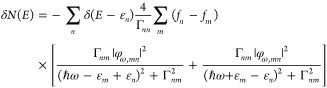
2

Evaluating this expression requires knowledge of the eigenfunctions and eigenvalues of Hamiltonian *H*_0_. Calculating these quantities using a diagonalization procedure becomes numerically challenging for large NPs of relevance to nanoplasmonic experiments.

### 2.4 Basis-Independent Population

To study large NPs, it is useful to express eq 2 in a basis-independent form. To achieve this, we first assume that Γ can be expressed as a function of the eigenenergies, i.e., Γ_*nm*_ = Γ(ε_*n*_,ε_*m*_). Then, following^37^ and introducing a double integral over ε and ε′ weighted by δ(ε – ε_*n*_)δ(ε′ – ε_*m*_), we find
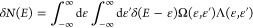
3with
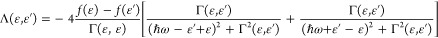
and
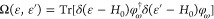


Note that Λ(ε,ε′) contains all of the information about relaxation processes through Γ. The Lorentzian form of the term inside the square brackets ensures energy conservation in optical transitions from ε to ε′ with a finite line width Γ(ε,ε′). The difference in Fermi functions ensures that only transitions from occupied states to unoccupied states are allowed. Ω(ε,ε′) is the energy-resolved optical transition matrix. Importantly, eq 3 factorizes the information about the statistics and relaxation processes in Λ(ε,ε′) and the information about the single-particle electron dynamics in Ω(ε,ε′). Typically, Ω(ε,ε′) is the most difficult object to calculate, but once it is obtained, any relaxation matrix can be studied without additional computational cost as a consequence of the factorization. Ω(ε,ε′) is calculated efficiently using KPM^25^ in the same way as in ref (37).

### 2.5 Numerical Considerations

The computation of Ω(ε,ε′) is the most numerically expensive part of the algorithm. It requires an average over random vectors and an expansion of the Dirac delta operators in the Chebyshev polynomials. Its time complexity is linear both in the number of random vectors *R* and in the number of atoms *N*, but quadratic in the number of polynomials *M*. Once Ω(ε,ε′) is obtained, it can be used to calculate the hot-carrier generation rate and the steady-state distribution for any temperature and relaxation matrix with little extra numerical effort. For our calculations, each Ω(ε,ε′) required *M* = 2000 Chebyshev polynomials and *R* = 300 random vectors. The simulations presented below were run in single-core mode on an AMD EPYC 7742 processor. For each random vector, the calculation of Ω(ε,ε′) took 2 min (requiring 3 GB of memory) for a small NP of 2 nm diameter and 5 h (62 GB of memory) for a large NP of 20 nm diameter.

## 3 Results and Discussion

In this section, we calculate the steady-state electron population in spherical Au and Ag NPs of different sizes. We also explore different simple models for the relaxation matrix but find that the gross features of the electron population do not depend on the model for the relaxation matrix.

### 3.1 Relaxation Matrix Model

The relaxation matrix Γ(ε,ε′) is one of the central objects in eq 3, and its functional form depends on the details of electron–electron and electron–phonon scattering mechanisms. While our approach allows a considerable amount of flexibility in choosing Γ, here we explore a few simple models to understand their effect on the steady-state electron population of spherical Au and Ag NPs. Following ref (13), we parameterize the matrix by the energy relaxation time τ_ε_ (ℏ/τ_ε_ = 1.3 meV for both Au and Ag) and the momentum relaxation time τ_p_ (ℏ/τ_p_ = 78 meV for Au and ℏ/τ_p_ = 20 meV for Ag). The momentum relaxation time is obtained from the Drude fit to the optical constants of Au and Ag obtained experimentally.^41^ The energy relaxation time estimates the electronic thermalization time and has considerable contributions from both electron–electron and electron–phonon scattering. Pump–probe experiments provide information about both these processes and place the corresponding times in the order of magnitude of 0.1–1 ps,^42−44^ depending on the experimental apparatus, NP sizes, and frequency at which the measurement was made (among other factors). For simplicity, we consider their collective effect to yield τ_ε_ ≈ 0.5 ps. In ref (13), Γ_*nn*_ = ℏ/τ_ε_ and Γ_*nm*_ = ℏ/τ_p_ if *m* ≠ *n*. To mimic this form for the relaxation matrix, we assign τ_ε_ to transitions that have very similar energies, that is, |ε_*n*_ – ε_*m*_| < 20 meV, and assign τ_p_ otherwise. This relaxation matrix is referred to as Γ_1_ and is given by
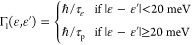


To test the sensitivity of our results to the numerical values of the relaxation times, we also present results for a relaxation matrix in which the value of τ_p_ is halved (denoted by Γ_2_) and a relaxation matrix in which the value of τ_ε_ is halved (denoted by Γ_3_).

**Figure 1:**
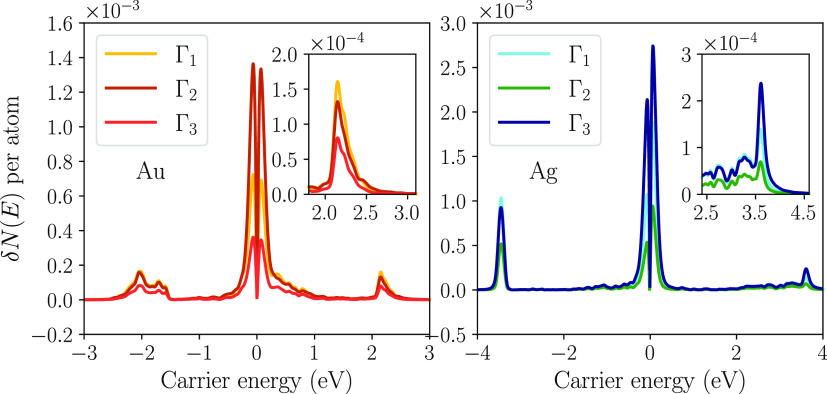
Steady-state hot-carrier population in spherical Au (left) and Ag (right) NPs with a diameter of 20 nm for three different relaxation matrices (Γ_1_, Γ_2_, and Γ_3_) at their corresponding LSPR (2.4 eV for Au, 3.5 eV for Ag) at room temperature (*T* = 298 K). The Fermi energy is set to zero, and the hole contribution to the hot-carrier population has been flipped in sign for clarity. The inset shows the high-energy peak of the hot-electron population.

Figure 1 compares the carrier population in Au and Ag NPs at their LSPR frequencies for the three relaxation matrices under an external electric field |**E**_ext_| = 8.7 × 10^–4^ V/nm, corresponding to an illumination power of 1 mW/μm^2^. Carriers with positive (negative) energy correspond to electrons (holes). The sign of the hole contribution is inverted for clarity. Transitions from the d band to the sp band generate hot holes and cold electrons in both metals, giving rise to a hole peak at the position of the d bands (−2 eV for Au, −3.5 eV for Ag) and a corresponding electron peak close to the Fermi level. Transitions in which both the initial and the final states derive from the sp band of the bulk material (also known as intraband transitions) are responsible for the electron peak at 2.2 eV in Au and the smaller electron peak at 3.5 eV in Ag. The large number of electrons and holes near the Fermi level is due to relaxation effects, which drive the hot electrons and holes back toward thermal equilibrium. See Section 3.2 for more details on interpreting these curves.

Comparing the results of the three different relaxation matrixes, we observe that the energy relaxation time mostly determines the overall magnitude of δ*N*: the populations obtained with Γ_1_ and Γ_3_ (which have the same momentum relaxation time) have the same shape but differ in their magnitude. This can be understood from eq 2 since 1/Γ_*nn*_ = τ_ε_/ℏ enters as an overall prefactor. In contrast, the shape of δ*N* is affected by the momentum relaxation time, especially around the Fermi level. However, we find that the qualitative features of the electron population are the same for all relaxation models, and we, therefore, present results only for Γ_1_ in the remainder of this paper.

### 3.2 Size Dependence

**Figure 2:**
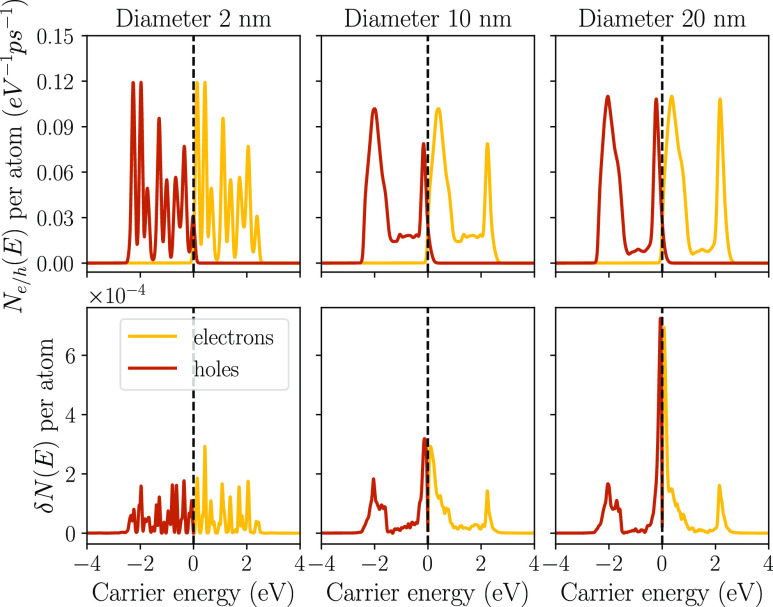
Hot-carrier generation rate (top) and population (bottom) of spherical Au NPs at the LSPR frequency (ℏω = 2.4 eV) for three different diameters at room temperature (*T* = 298 K).

In this section, we explore the effect of the NP size on the steady-state population. Figure 2 shows the rate of carrier generation in Au NPs, obtained using the method from ref (37) as well as the electron and hole populations inside the NP after relaxation at the LSPR frequency ℏω = 2.4 eV.

**Figure 3:**
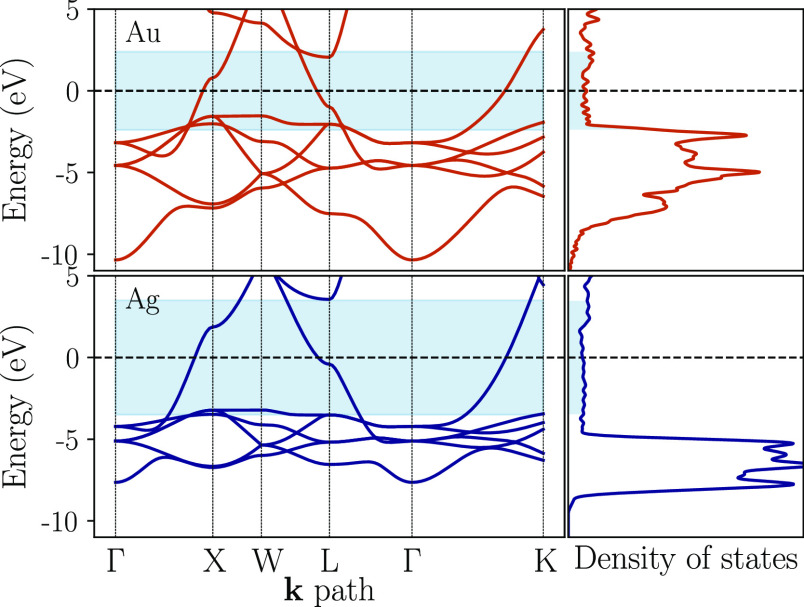
Band structure and density of states of Au and Ag, using the tight-binding model from ref (40). The state that can participate in optical transitions at the LSPR frequency lies within the blue rectangle, and the Fermi energy is set to zero.

The hot-carrier generation rates have contributions from both momentum-conserving (direct) and nonconserving (indirect) optical transitions. Direct transitions can be understood in terms of gold’s band structure (see top panel of Figure 3). There are a large number of transitions arising from the d bands at *E* = −2 eV, giving rise to a large number of hot holes at −2 eV and cold electrons at 0.4 eV, close to the Fermi level. A similar situation occurs at *E* = 2.2 eV at the onset of an sp band. These transitions generate many hot electrons but relatively cold holes at −0.2 eV. Indirect transitions do not conserve momentum, and the typical scale of momentum transferred is inversely proportional to the NP diameter.^14^ For this reason, the relative importance of indirect transitions increases as the NP size decreases. For very small NPs (see results for 2 nm diameter), the generation rate exhibits a molecule-like behavior with many discrete peaks arising from quantum confinement effects.

The steady-state hot-carrier population exhibits a similar peak structure as the generation rate, but the relative height of the peaks is different compared to the generation rate: after the carriers are generated, they relax via electron–electron and electron–phonon interactions, losing energy and approaching the Fermi level. For example, electrons are constantly generated at *E* = 2.2 eV, contributing to the *E* = 2.2 eV peak in the electron population (see the bottom panel of Figure 2). However, the electrons constantly relax back to the Fermi energy and contribute to the peak near *E* = 0 eV. A similar picture holds for the holes generated at *E* = −2 eV.

To quantitatively study the number of high-energy electrons (δ*N*_e_^HE^) and holes (δ*N*_h_^HE^) in the steady-state population, we evaluate
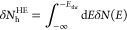
4
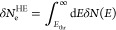
5where *E*_thr_ = 1.5 eV denotes a threshold energy. The value of the threshold energy is chosen such that the contribution from the high-energy peaks in δ*N*(*E*) is captured.

**Figure 4:**
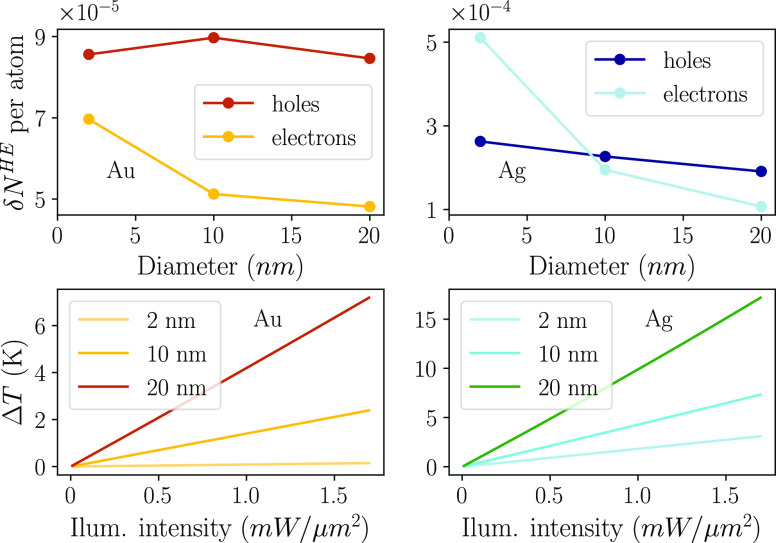
Top panels: number of high-energy electrons and holes in the steady-state population. In Au, high-energy carriers are defined as having an energy larger than 1.5 eV relative to the Fermi level. In Ag, the corresponding energy threshold is 2.0 eV. Bottom panels: increase in electronic temperature as a function of external illumination strength for different NP diameters.

Figure 4 (top left) shows that significantly more energetic holes are available in the steady-state population than energetic electrons, in particular for larger NPs. This asymmetry can be understood from the amount of available interband transitions. Figure 3 (top) shows that there is a much larger overlap of the LSPR zone with the d bands at −2 eV than with the sp band at 2 eV. This asymmetry becomes more pronounced as the relative number of surface-enabled intraband transitions diminishes for larger NPs.

**Figure 5:**
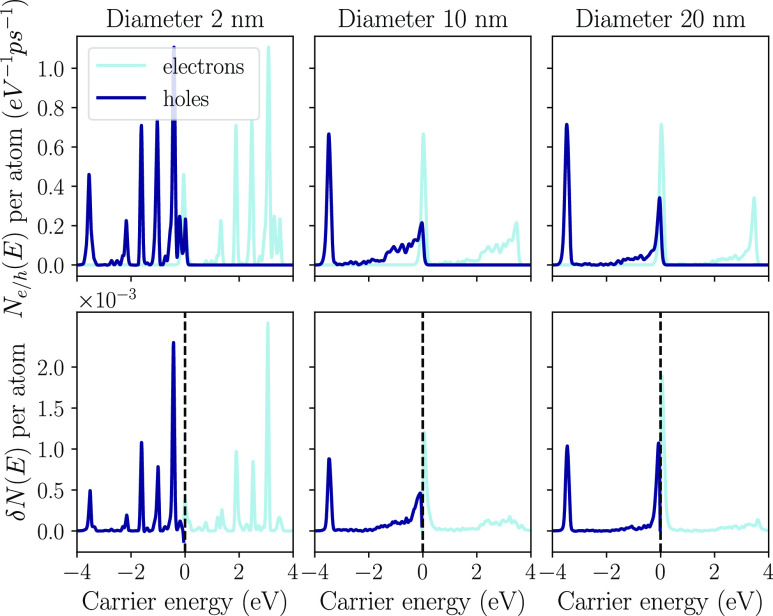
Hot-carrier generation rates (top) and populations (bottom) for three different sizes of Ag NPs at the LSPR frequency (ℏω = 3.5 eV) at room temperature (*T* = 298 K).

Next, we repeated the analysis for Ag NPs. Ag has a qualitatively similar band structure to Au (see bottom panel of Figure 3), but both the d bands and the unoccupied sp bands are further away from the Fermi level. The LSPR frequency is ℏω = 3.5 eV instead of ℏω = 2.4 eV. Compared to Au, the LSPR region has a smaller overlap with these bands in Ag (see Figure 3), so we expect the high-energy peaks to be sharper in both the generation rate and the steady-state population due to the smaller number of available transitions. This is indeed observed in Figure 5. The pronounced peak in the hot-hole generation at −3 eV persists in the hole population, but the sharp peak at 3.5 eV in the hot-electron generation rate almost disappears after relaxation effects are taken into account. In its place, a relatively uniform and broad distribution of hot electrons appears.

Because of this sensitivity to the availability of compatible interband transitions in the hot-electron sector, there is a strong size dependency in the number of hot electrons δ*N*_e_^HE^ to NP size; see Figure 4 (top right). In contrast, the number of high-energy holes is almost constant. Note that an energy threshold of *E*_thr_ = 2.0 eV was used in eqs 4 and 5 for Ag NPs.

### 3.3 Electronic Temperature

The NP’s temperature is a key ingredient in explaining many chemical phenomena, and the electronic temperature can be estimated from energy dissipation with the heat equation^45^ or by finding the solution to Boltzmann’s equation in a steady state.^10^ Our formalism also provides a way to compute the temperature of the electrons inside the NP via its connection to the Fermi function. In equilibrium, the electronic temperature *T* can be determined from the slope of the Fermi–Dirac population at the Fermi level (which we set to zero here), i.e., *f*′(0) = −1/(4*k*_B_*T*) (with *k*_B_ being the Boltzmann constant). Upon illumination, the electron population is no longer determined by a Fermi–Dirac distribution but instead given by *N*(*E*) = ∑_*n*_*f*(ϵ_*n*_)δ(*E* – ϵ_*n*_) + δ*N*(*E*), see eq 2. Note that near the Fermi level, we have ∑_*n*_*f*(ϵ_*n*_)δ(*E* – ϵ_*n*_) ≈ *f*(*E*)*D*(0) with *D*(*E*) = ∑_*n*_δ(*E* – ϵ_*n*_) denoting the density of states, which varies slowly near the Fermi level; see Figure 3.

To extract an electronic temperature from *N*(*E*), we assume that it behaves like an equilibrium population with an elevated temperature *T*′ near the Fermi level, i.e., *N*(*E*) ≈ *f*_*T*′_(*E*)*D*(0) (where we now explicitly write the temperature as a subscript to the Fermi–Dirac function). Near the Fermi level, we thus have
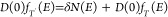


The new temperature of the NP is then found to be
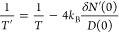


The increase in electron temperature as a function of the illumination intensity is shown in Figure 4 (bottom). For the range of illumination intensities in Figure 4, the temperature increase is proportional to the illumination intensity, and the temperature increase does not exceed 17 K. Larger NPs exhibit a larger increase in temperature compared to smaller NPs as there is a larger portion of electrons and holes close to the Fermi energy (see Figures 2 and 5). Ag NPs show a much higher temperature increase than Au NPs for the same field strength. This can be understood by observing that the electric field inside the NP is much larger in Ag than in Au at their respective LSP frequencies, as can be seen from the prefactor α(ω) in eq 1. The resulting perturbation, which is proportional to α(ω), is therefore much larger in Ag. As a consequence, an Ag NP has a much larger absorption cross-section than an Au NP (by a factor of 6).

## 4 Conclusions

We have developed an atomistic approach to calculate the steady-state hot-carrier population in spherical Au and Ag NPs containing hundreds of thousands of atoms, thus enabling the modeling of large NPs of relevance to experimental studies. This was achieved by expressing the population in a basis-independent way using KPM. Relaxation processes are included in this formalism through an energy-dependent relaxation matrix Γ(ε,ε′). We explored different simple models for the relaxation matrix but found that the qualitative predictions do not depend on this choice. The hot-carrier populations of Ag and Au NPs exhibit similar features, but the number of highly energetic electrons in Ag NPs was found to be highly sensitive to the NP size. We explain this observation in terms of Ag’s band structure, which features an occupied band whose energy relative to the Fermi level is almost exactly the same as Ag LSP energy. Furthermore, the increase of the electron temperature in the NP is estimated, and it is found that Ag NPs exhibit a much larger increase as a consequence of a considerably larger absorption cross section in the Ag NP. The approach developed in this work can be used to study the hot-carrier population in NPs of complex shapes and compositions. This will be the focus of future work.
